# The Sanitation Hygiene Infant Nutrition Efficacy (SHINE) Trial: Protocol for school-age follow-up

**DOI:** 10.12688/wellcomeopenres.19463.1

**Published:** 2023-07-14

**Authors:** Joseph D. Piper, Clever Mazhanga, Marian Mwapaura, Gloria Mapako, Idah Mapurisa, Tsitsi Mashedze, Eunice Munyama, Maria Kuona, Thombizodwa Mashiri, Kundai Sibanda, Dzidzai Matemavi, Monica Tichagwa, Soneni Nyoni, Asinje Saidi, Manasa Mangwende, Dzivaidzo Chidhanguro, Eddington Mpofu, Joice Tome, Batsirai Mutasa, Bernard Chasekwa, Melanie Smuk, Laura E. Smith, Handrea Njovo, Chandiwana Nyachowe, Mary Muchekeza, Kuda Mutasa, Virginia Sauramba, Lisa F. Langhaug, Naume V. Tavengwa, Melissa J. Gladstone, Jonathan C. Wells, Elizabeth Allen, Jean H. Humphrey, Robert Ntozini, SHINE Follow-up team, Andrew J. Prendergast

**Affiliations:** 1 Blizard Institute, Queen Mary University of London, London, England, UK; 2 Zvitambo Institute for Maternal and Child Health Research, Harare, Harare Province, Zimbabwe; 3 Cornell University, Ithaca, New York, USA; 4 Ministry of Health and Child Care, Harare, Zimbabwe; 5 Institute of Translational Medicine, University of Liverpool, Liverpool, England, UK; 6 Population Policy and Practice Research and Teaching Department, UCL Great Ormond Street Institute of Child Health, London, UK; 7 London School of Hygiene and Tropical Medicine, London, WC1E 7HT, UK; 8 Department of International Health, Johns Hopkins Bloomberg School of Public Health, Johns Hopkins University, Baltimore, Maryland, USA

**Keywords:** Child, cognition, fitness, body composition, HIV, nutrition, IYCF, WASH

## Abstract

**Background**: There is a need for follow-up of early-life stunting intervention trials into childhood to determine their long-term impact. A holistic school-age assessment of health, growth, physical and cognitive function will help to comprehensively characterise the sustained effects of early-life interventions.

**Methods:** The Sanitation Hygiene Infant Nutrition Efficacy (SHINE) trial in rural Zimbabwe assessed the effects of improved infant and young child feeding (IYCF) and/or improved water, sanitation and hygiene (WASH) on stunting and anaemia at 18 months. Among children enrolled to SHINE, 1,275 have been followed up at 7-8 years of age (1,000 children who have not been exposed to HIV, 268 exposed to HIV antenatally who remain HIV negative and 7 HIV positive children). Children were assessed using the School-Age Health, Activity, Resilience, Anthropometry and Neurocognitive (SAHARAN) toolbox, to measure their growth, body composition, cognitive and physical function. In parallel, a caregiver questionnaire assessed household demographics, socioeconomic status, adversity, nurturing, caregiver support, food and water insecurity. A monthly morbidity questionnaire is currently being administered by community health workers to evaluate school-age rates of infection and healthcare-seeking. The impact of the SHINE IYCF and WASH interventions, the early-life ‘exposome’, maternal HIV, and contemporary exposures on each school-age outcome will be assessed. We will also undertake an exploratory factor analysis to generate new, simpler metrics for assessment of cognition (COG-SAHARAN), growth (GROW-SAHARAN) and combined growth, cognitive and physical function (SUB-SAHARAN). The SUB-SAHARAN toolbox will be used to conduct annual assessments within the SHINE cohort from ages 8–12 years.

**Ethics and dissemination:** Approval was obtained from Medical Research Council of Zimbabwe (08/02/21) and registered with Pan-African Clinical Trials Registry (PACTR202201828512110, 24/01/22). Primary caregivers provided written informed consent and children written assent. Findings will be disseminated through community sensitisation, peer-reviewed journals and stakeholders including the Zimbabwean Ministry of Health and Child Care.

## Article summary

- This protocol describes the follow-up of school-age children after receiving early-life IYCF and WASH interventions in rural Zimbabwe.
- A comprehensive measurement of the child’s health, growth, body composition, physical and cognitive function has been performed.
- Analysis of the ‘exposome’, including maternal HIV status, will identify the early-life factors that shape school-age physical and cognitive function.
- Capitalizing on the randomized trial design will allow the long-term effects of IYCF and WASH on child health outcomes to be estimated.
- Limitations include the re-enrolment of only a subgroup of children from one of the two original study districts; the low numbers of children living with HIV; and the long period between early-life randomized interventions and school-age outcome assessment.

## Introduction

Children are defined as “stunted” when their height-for-age Z-score (HAZ) is more than two standard deviations below the World Health Organization (WHO) reference standard, but linear growth faltering also affects many children who have not yet fallen below this cutoff ^1^. Stunting is associated with increased mortality, poorer school performance, lower adult earnings and long-term chronic disease ^2^. Stunting affects 22% (149 million) of children under 5 years old ^3^, while up to 250 million children are at risk of not reaching their developmental potential ^4^. This makes stunting a global health priority, although its causes, longer term impact and response to interventions remain poorly understood.

**Figure 1.**
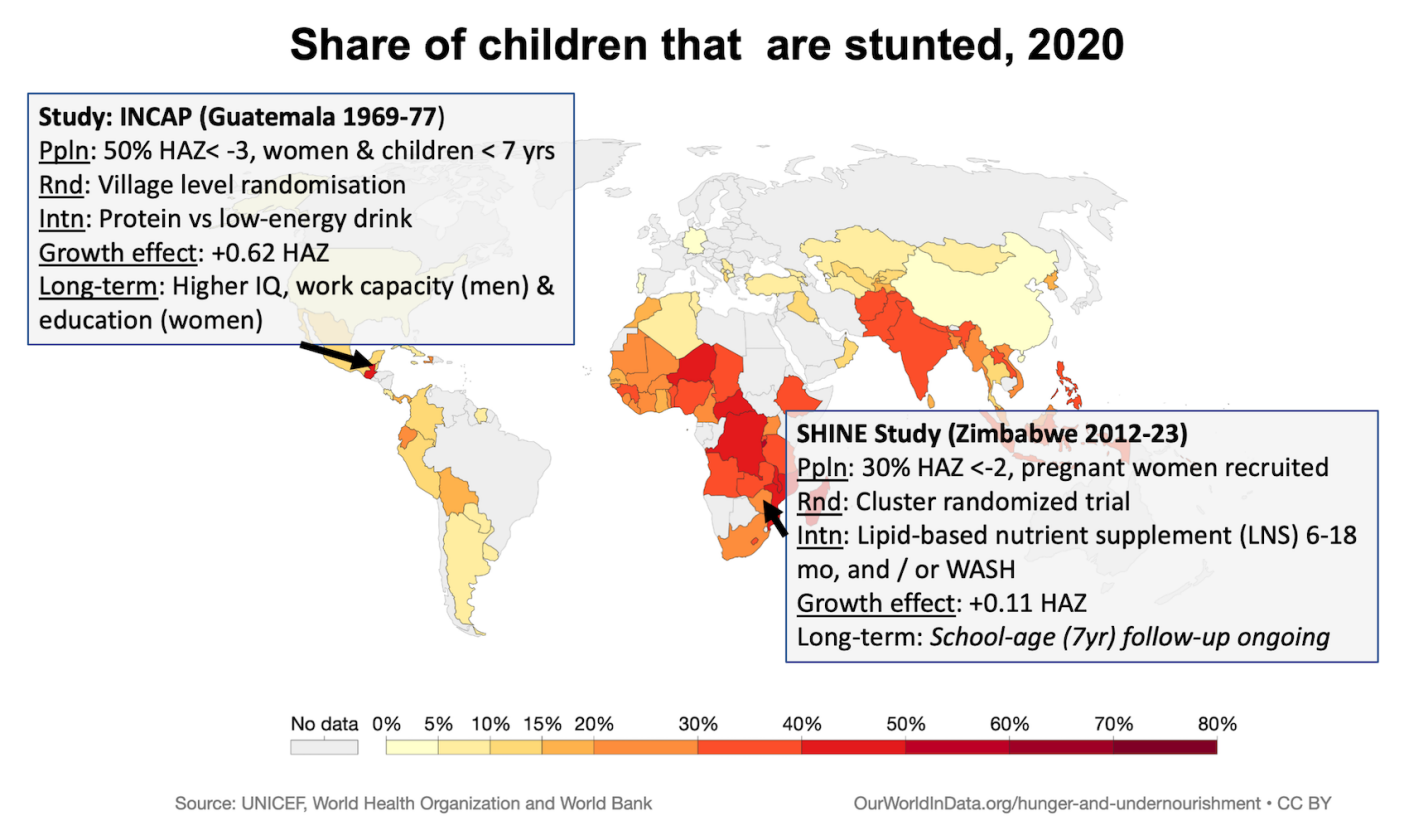
Proportion of children that are stunted (UNICEF, WHO and World Bank, kindly reproduced from ‘Our World in Data’). Inset details describe the landmark INCAP study and the proposed SHINE study, both of which provide long-term follow-up of a nutrition intervention. Data from the landmark INCAP study in Guatemala 50 years ago indicate that early-life improvements in nutrition can confer long-term benefits for cognition. Children receiving additional nutrition by age 2 years initially had only modest effects on neurodevelopmental scores; however, in long-term follow-up, those who had received the nutrition intervention had higher IQ scores, greater work capacity and earnings (among men) and greater schooling (among women) ^4^. The INCAP study was conducted at a time when global stunting prevalence was much higher (1969–1977): 50% of the study population had HAZ<-3.0, while currently worldwide 22% children have HAZ <-2.0. Furthermore, the impact of the intervention on linear growth was much greater than that seen in trials of complementary feeding interventions over the past 20 years (+0.62 HAZ compared to +0.11 HAZ). Thus, although the Guatemala trial suggests that complementary feeding interventions can have substantial long-term physical and neurodevelopmental benefits, it does not reflect today’s situation in which Africa has the highest stunting prevalence, severe stunting is relatively rare but moderate stunting is 20–40%, and the average impact of interventions on HAZ is only 0.1-0.2. UNICEF, United Nations Children's Fund; WHO, World Health Organization; INCAP, Institute of Nutrition of Central America and Panama; SHINE, Sanitation Hygiene Infant Nutrition Efficacy; HAZ, height-for-age Z-score; Ppln, population; Rnd, randomisation technique; Intn, Intervention; WASH, water, sanitation and hygiene.

Given the ongoing global consequences of stunting, long-term follow-up studies reflecting contemporary conditions, geography and interventions for stunting are urgently needed to inform robust, cost-effectiveness analyses and advocacy of IYCF and WASH policies and programming ( Figure 1).

**Figure 2.**
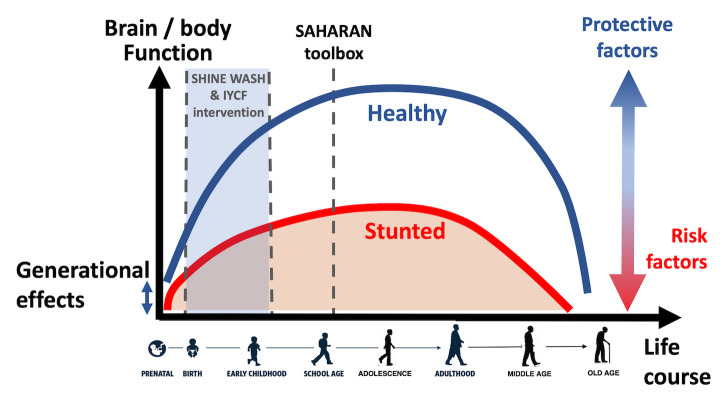
The life course approach to child growth and development (reproduced from Child Health for all, 6 ^th^ Edition with permission from Oxford University Press). Healthy (blue) and stunted (red) trajectories are shown, illustrating that lifelong health and function are particularly affected by early-life conditions, as well as risk and protective factors throughout life. This applies to linear growth, physical function and health as well as cognitive function and mental health. IYCF, infant and young child feeding.

The lack of long-term data evaluating *combined* neurodevelopment, physical fitness and growth has led to a call for further studies that measure a range of outcomes ^5, 6^. A holistic measurement is vital to understand school-age trajectories across different functional domains and the impact of risk and protective factors and potential interventions ( Figure 2). At school-age, it becomes easier to undertake more detailed measures of cognitive development, including school performance, executive function and socio-emotional behaviour.

### Aims and objectives

This study is undertaking long-term follow-up of a cohort of children enrolled in the Sanitation Hygiene Infant Nutrition Efficacy (SHINE) trial in rural Zimbabwe. SHINE was a 2x2 factorial cluster-randomized trial across two contiguous districts in rural Zimbabwe between 2012–17, designed to test the independent and combined effects of improved infant and young child feeding (IYCF) and improved water, sanitation and hygiene (WASH) on child height-for-age Z score (HAZ) and haemoglobin at 18 months of age. All outcomes were stratified by maternal HIV status. Detail of the trial design ^7^ and primary results ^8– 11^ are published. In brief, SHINE found that the IYCF intervention modestly improved linear growth, reducing stunting prevalence by 20%, while the WASH intervention had no significant effect. Children who have been exposed to HIV and remain HIV-negative (HIV-exposed and uninfected, CHEU) had a higher risk of stunting and impaired neurodevelopment than children who have not been exposed to HIV (HIV-unexposed, CHU), but also intriguing evidence of increased responsiveness to early-life interventions ^10, 11^.

Long-term follow-up of this cohort now enables us to address: i) the effects of the randomised IYCF and WASH interventions on school-age growth, body composition, cognitive and physical function; ii) the impacts of early-life stunting and a range of other exposures (the ‘exposome’) on school-age growth, body composition, cognitive and physical function; and iii) the differences in school-age growth, body composition, cognitive and physical function between CHEU and CHU. Therefore, the objectives are summarised as follows:

1) Evaluate the impact of the IYCF intervention on school-age growth, health, physical and cognitive function (both for children who have been exposed to HIV and those who have not been exposed).
2) Evaluate the impact of the WASH intervention on school-age growth, health, physical and cognitive function (both for children who have been exposed to HIV and those who have not been exposed).

3) Evaluate the relationship between the exposome during the first 1,000 days and school-age growth, health, physical and cognitive function, including:
  a) Early-life length-for-age Z-score, and categorical definitions of stunting (LAZ<-2) by 1 month of age (early stunting) and by 18 months of age (late stunting).
  b) Environmental factors, including socioeconomic status, household composition, demographics, maternal education, household adversities, food and water insecurity.
  c) Pregnancy exposures including maternal caregiving capabilities, nutritional status and depression.
  d) HIV exposure in pregnancy.

4) Evaluate the relationship between current environmental, schooling, nurturing and care-giving practices and school-age growth, health, physical and cognitive function.
5) Develop and deploy three novel, holistic and succinct *outcome* metrics to assess school-age growth, health, physical and cognitive function.

## Methods

SHINE enrolled pregnant women from 210 clusters ^7, 8^, each representing the catchment area of one to four community health workers (CHW) and covering a geographic area of 8,232 km ^2^. For this long-term follow-up study, 1,275 children born to these women from 105 clusters in Shurugwi district were enrolled. A total of 1,000 children who originally participated in SHINE and were aged between 7–8 years during the period of enrolment and have not been exposed to HIV (CHU) were randomly selected by a computer from clusters by the data team. For those who had moved away or were otherwise lost to follow-up, randomised replacements within the SHINE cohort were selected to ensure 1,000 children. All children exposed to HIV were invited to enrol, with 268 CHEU enrolled in total; seven children found to be living with HIV were excluded from the analysis, and will form the basis of a separate case report, given their low number. Children no longer resident in Shurugwi, with unknown maternal pregnancy HIV status, or outside the age window were ineligible. There are no new interventions in the long-term follow-up study.

### Participant and public involvement

School-age participants and their caregivers were not involved in designing the protocol or selecting outcomes. However, they were involved in design and implementation of the questionnaires and tools. All tests to measure school-age child growth, physical and cognitive function were discussed with the District Health Executive and community leaders. All questionnaires underwent cognitive interviewing with community members who suggested alterations and feedback. Tools were then pre-tested with local families to ensure acceptability, with feedback sought from children and caregivers. At the start of the follow-up study, sensitisation of the local leadership and community members was undertaken by the study team, together with the screening of a community-made film about the SHINE trial, which explored their previous involvement and experiences of research. CHWs performed role-plays to explain the purpose, consent procedures and practicalities of the visit to participating families and the community. Once the family had received information and expressed an interest in joining, a mutually convenient date was booked for an assessment.

### Screening, consent, assent and assessments

Assessments were conducted by data collectors (DCs), who were primary care nurses extensively trained in study procedures. On the day of the assessment, the CHW introduced a pair of DCs (DC1 and DC2) to the household. Screening was undertaken to ensure the original SHINE child and primary caregiver were present. The verification for the primary caregiver (usually the mother) and child was mainly through the birth certificate, identity card and other papers such as the child’s Ministry of Health baby health card, as well as noting any evidence of previous recruitment into the SHINE trial ( *e.g.*, family showing previous consent documents or a SHINE latrine at the household). Once both DCs verified that this was the correct child, one to two tents were pitched in or close to the household. Households were usually single-family dwellings surrounded by subsistence farmland and the mean distance between houses in SHINE was 82.6 m ^8^, ensuring the tents were sufficiently isolated for confidentiality during informed consent and data collection. Written informed consent was obtained from the primary caregiver, and assent from the child, following a demonstration of the tools used to conduct the measurements. The primary caregiver was usually the mother, but it was the child born within the SHINE trial who is the focus of this follow-up study. Hence if the mother was not available (for example the mother had died or moved away), the primary caregiver was defined as the adult most responsible for the child’s care and completed the caregiver questionnaire.

DC1 performed the caregiver questionnaire in a pre-specified order, while DC2 conducted the cognitive, physical and anthropometry assessments in a separate tent to minimize distractions, after building rapport with the child. The child was fed before the start of the tests (either by the family or the DC’s who provided an additional snack of biscuits and Maheu, a traditional maize-based drink). The child was then given further regular breaks and snacks (also biscuits and Maheu) to reduce tiredness. All tests had standardized explanations and demonstrations to ensure appropriate understanding, and were translated into Shona or Ndebele—the local languages spoken in the district. The DCs remained blinded to the original trial allocation and the maternal HIV status throughout the visit. At the end of the visit, DC1 opened a sealed envelope to check the maternal HIV status in the original trial, which then determined whether questions were asked on HIV treatment or if HIV testing was appropriate. At the end of the visit, a small gift was given to the child and caregiver to thank them for their time comprising: a pencil, two pens, exercise book, story book, bar of soap, jar of peanut butter and two litre bottle of cooking oil. The start and end time and temperature at the beginning and end of the assessments was also recorded.

### HIV testing and referrals

All primary caregivers were offered HIV testing at the end of the visit unless they were already known to be living with HIV or had a documented negative result in the previous three months. If the mother did not have HIV, the child was not tested. If the mother was living with HIV, declined testing or was not available, the child was offered HIV testing with age-appropriate assent using role-plays. The Determine HIV-1/2 rapid test (Abbott) was used for initial testing; positive results were repeated using the HIV 1/2 Stat-Pak rapid test (Chembio). The option of dried blood spot testing was also available for participants who preferred not to be tested in real-time in the homestead. Referrals were made to local clinics for mothers or children testing positive for HIV or for other conditions found on visiting which necessitated a referral, as defined by a SOP.

### Outcome measures

All assessment tools were integrated into a single assessment lasting 4–5 hours, using the School Age Health, Activity, Resilience, Anthropometry and Neurocognitive (SAHARAN) toolbox, which was developed, refined and piloted by our team ^12^. Briefly, SAHARAN includes a caregiver questionnaire, child questionnaire and direct tests undertaken with the child, focused on three domains: cognitive function, growth and body composition, and physical function. The primary outcome of the long-term follow-up study is cognitive function, assessed by the mental processing index (MPI)—the total score from eight subtests of the Kaufman Assessment Battery for Children 2 ^nd^ edition (KABC-II) ^13^. The subtests measure four domains of cognitive processing across learning, planning, simultaneous and sequential memory. All other outcomes are secondary outcomes and shown in Table 1– Table 3.

**Table 1.** Cognitive outcomes to be measured, including the primary outcome (MPI) and secondary outcomes. MPI, mental processing index; KABC-II, Kaufman Assessment Battery for Children 2 ^nd^ edition.

	MARKER	MEASURE	OUTCOMES	RATIONALE
**Cognitive** **Function** **(120 mins** ** including** ** KABC-II)**	**Kaufman Assessment ** **Battery for Children**	**Cognitive processing**	**Primary outcome: Mental ** **processing index (MPI)**	**Overall measure of ** **cognitive function**
			Secondary outcomes: KABC-II domain scores, individual subtest scores	Short & long-term memory, planning, problem-solving, sequential memory
	**School Achievement Test**	Academic	Total score, Subtest scores ( *numeracy, reading, writing)*	Literacy & numeracy
	**Fine motor**	Shortest time to complete finger tapping sequence	Time for dominant hand, non- dominant hand, and average between both hands,	Fine motor
	**Plus-EF Tablet test**	Executive Function	Overall score, individual subtest scores, reaction time	Executive function
	**Child socio-emotional** ** questionnaire**	Home support	Total score, sub-score removing food insecurity question	Child’s own perspective on home support
	**Washington Group Child ** **function module (asked in ** **caregiver questionnaire)**	Disability screening, including vision and hearing	Overall score, disability, learning, mental health subscale	Child functional abilities
	**Strength and Difficulties ** **Questionnaire (SDQ, asked ** **in caregiver questionnaire)**	Socioemotional function	SDQ total score and subtest scores	Behaviour

**Table 2.** Growth and body composition outcomes. MUAC, mid upper-arm circumference; HAZ, height-for-age Z-score; WAZ, weight-for-age; BMI, body mass index.

	MARKER	MEASURE	OUTCOMES (secondary)	RATIONALE
**Body** ** composition (20** ** mins)**	**Bio-impedance analysis (BIA)**	Impedance of tissues	Lean mass index, Phase angle, Impedance index, Reactance, Resistance	Quality of growth, metabolic health
	**Knee-heel length**	Tibial growth	Median Knee-heel length	Prioritization of growth
	**Triceps, scapular, supra-iliac, ** **calf skinfolds**	Subcutaneous fat	Sum of skinfolds, Individual skinfolds, Peripheral: central skinfolds,	Subcutaneous fat: peripheral *vs.* central, metabolic health
**Anthropometry** ** (15 mins)**	**Height, weight**	Growth	HAZ, WAZ, BMI	Growth, nutritional status, Metabolic health
	**Head circumference**	Brain volume	Head circumference	Prioritization of growth
	**Waist and hip circumference**	Abdominal size	Waist circumference, hip circumference	Nutritional status, metabolic health
	**Calf circumference, MUAC**	Peripheral fat & muscle	Calf circumference, MUAC	Quality of growth

**Table 3.** Physical function outcomes.

	MARKER	MEASURE	OUTCOMES (secondary)	RATIONALE
**Physical** **Function** ** (30 mins)**	**Grip strength (a)**	Lean muscle both hand	Highest grip strength, Standardised grip strength **(a)** Dominant and non-dominant hand strength,	Lean muscle: hand
	**Broad jump (b)**	Truncal muscles	Maximum distance, standardised distance ( **b)**	Lean muscle: leg
	**20m Beep test (c)** **(composite score =** ** standardised a+b+c)**	Physical Fitness,	Shuttle run test level Standardised shuttle run test level: **(c)** ***Composite standardised score =*** *** a+b+c***	Stamina, Overall composite score
	**Haemoglobin**	Anaemia	Hb	Physical fitness
	**Blood pressure (BP)**	Fitness	Resting Systolic & diastolic BP, Pulse pressure, systolic & diastolic BP & pulse pressure 1 minute after exercise; Post-exercise difference between 1 ^st^ and 5 ^th^ BP systolic & diastolic measurement	Cardiovascular fitness

***Support and supervision*.** Ongoing support and monitoring were provided by a senior research nurse and a doctor, with 6-monthly standardisation exercises. Additional training was provided for any DCs below a set threshold in anthropometry or cognitive measurement, derived from the intra- and inter-observer technical error of measurement for continuous measurements ( *e.g.*, anthropometry) ^14^ and intraclass correlation coefficient (ICC) for discrete measurements ( *e.g.*, cognitive test scores).

***Measurement of illness and healthcare-seeking behaviour*.** The incidence of common infections is measured by prospective surveillance. The CHW visits families 4-weekly (with a visit window of 3 to 5 weeks) to conduct a 7-day illness recall questionnaire to capture episodes of fever, cough, diarrhoea, skin, ear or mouth infections or other illnesses, and associated health-seeking behaviours.

### Sample size

The primary outcome is the total KABC-II from all subtests, called the Mental processing index (MPI). The raw subtest scores are adjusted by age into 4-month blocks. A total of 1,000 children (500 IYCF *vs.* 500 non-IYCF) were assessed, providing 86% power to detect a 0.2 standard deviation difference in MPI between IYCF and non-IYCF arms with alpha 0.05, assuming intra-cluster correlation of 0.05 and sampling from 100 clusters. This will allow the exploration of the difference in IQ scores observed at 3–7 years of age among children followed-up in the Institute of Nutrition of Central America and Panama (INCAP) study, and is also the approximate magnitude of socio-emotional difference recently shown with a similar small-quantity lipid-based nutrient supplement trial ^15^. Since data collection was undertaken directly after consent and enrolment, no adjustment for lost to follow-up was required. The SHINE 2x2 factorial design also enables 500 WASH *versus* 500 non-WASH children to be assessed as a secondary outcome. For CHEU, the sample size is 268, which allows exploration of the association between the treatment arms (IYCF *vs.* non-IYCF, and WASH *vs.* non-WASH) for secondary outcomes.

### Data management

DCs electronically collected the SAHARAN questionnaire and observation data onto password-protected Android tablets (Samsung Galaxy Tab A) using the Open Data Kit (ODK) platform ( https://opendatakit.org/). The ODK forms were programmed with skip patterns, expected data ranges and free text sections to report any issues with data collection. Back-up paper forms were also provided in case of tablet failure. Data were manually checked by the Project Lead and Field Data Officer and then uploaded onto an ODK Aggregate Server, and stored on an SQL Server database at the end of each working day. All data were collected using a unique participant identifier (PID) to maintain confidentiality. A CC-BY license will be applied. Given this was a long-term follow-up of a cluster randomised trial (with the interventions previously completed), a data monitoring committee or interim analysis was not needed.

### Statistical analysis

A Statistical Analysis Plan (SAP) designed to give transparency to possible influential statistics decisions for the study was finalised prior to unblinding and initiation of analyses and is available as *Extended data* ^16^.

 Reporting of results will follow the guidelines established in the extended CONSORT guidance for cluster-randomized trials ^17^.

We used the SPIRIT checklist ^16^ when writing our report given that the SHINE cluster randomised trial primary outcome at 18 months has been completed. The analyses are described below according to the objectives ^18^.

***Effect of IYCF and WASH on outcomes (Objectives 1 & 2)*.** We will capitalize on the 2x2 factorial trial design to evaluate the IYCF and WASH interventions as two trials run in the same population, stratified by maternal HIV status. For the analysis of IYCF as the primary outcome, we will therefore combine the two IYCF-containing trial arms (IYCF alone and IYCF+WASH) and compare them to the two non-IYCF arms (WASH alone and standard-of-care). For the analysis of WASH as a secondary outcome, we will combine the two WASH-containing trial arms (WASH alone and IYCF+WASH) and compare them to the two non-WASH arms (IYCF alone and standard-of-care). The effect of IYCF and WASH on each outcome (as defined in Table 1 to Table 3) will be evaluated using generalized estimating equations (GEE) with an exchangeable working correlation structure to account for within-cluster correlation, assuming no interactions between randomised interventions. All analyses will account for the nature of the distribution of the outcome and results will be presented as appropriate effects sizes (mean difference between groups and risk ratios) with a measure of precision (95% confidence intervals). Analyses will be by intention-to-treat at the child level, according to the mother’s assigned study arm based on her residence at the time of her enrolment into SHINE, regardless of subsequent moving or adherence to the interventions. The hypothesis test will be 2-sided and a significance level of 5% will be used.

The primary analysis will be unadjusted. A secondary adjusted analysis will account for original trial stratification factors and other possible confounders as defined in the SAP. Since we are performing a large number of tests, we will base our conclusions on associations observed in outcome groupings, and not on p-values. The generalisability of the sample will be assessed in comparison to the original SHINE cohort. If evidence of a large difference is seen, then weights will be applied as a sensitivity analysis to explore inference effect.

**Figure 3.**
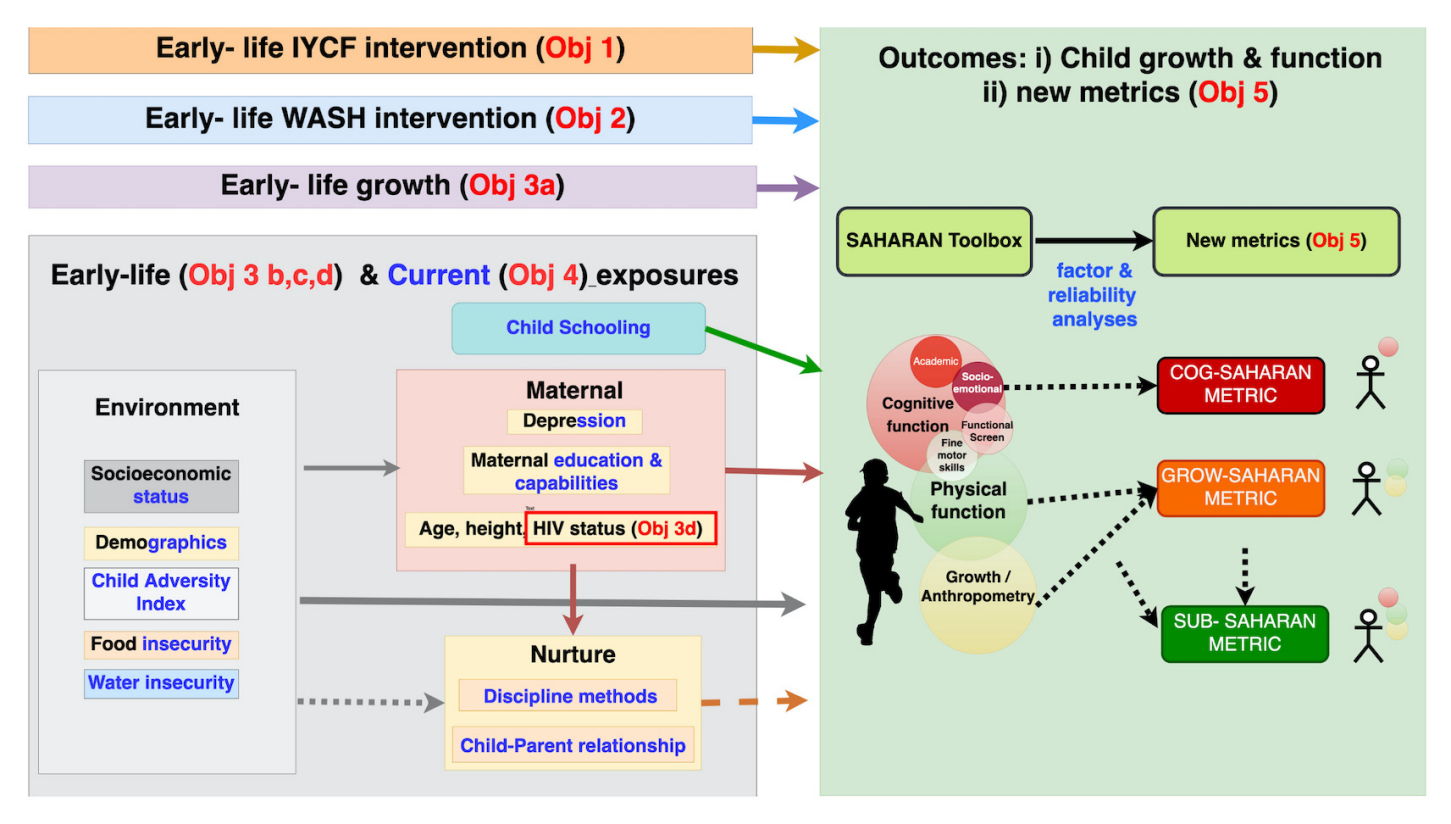
Conceptual framework of objectives with exposure and outcome variables. Exposures are split into environmental, schooling, maternal and nurturing domains. Early-life exposures are described in black and contemporary exposures in blue text. Those exposures that were measured in both early-life and contemporary are written in black and blue text. Outcomes are based on the SAHARAN toolbox to provide school-age child growth, health and function. These outcomes will also be analysed in the standard of care arm to provide new cognitive (COG-SAHARAN), Growth (GRO-SAHARAN) and overall (SUB-SAHARAN) metrics. SAHARAN, School-Age Health, Activity, Resilience, Anthropometry and Neurocognitive; IYCF, infant and young child feeding; WASH, water, sanitation and hygiene.

***Evaluating early-life growth and exposures on school-age function (Objectives 3a, b, c)*.** As secondary outcomes, we will explore the effects of early stunting (by 1 month of age) and late stunting (by 18 months of age) on each outcome at 7 years of age, and the effect of LAZ as a continuous variable at 1 and 18 months. We will also perform a principal components analysis on the 7 year outcomes to reduce the number of outcome variables into a smaller set of functional biological domains that best represent the most informative combination of outcomes. Early-life factors can be grouped into environmental ( *e.g.*, socioeconomic status, food and water insecurity, household adversities), maternal ( *e.g.*, maternal education and depression) and nurturing factors (caregiving) ( Figure 3). For the early-life exposome, we will use least absolute shrinkage and selection operator (LASSO) for Generalised Estimating Equations (GEE) to quantify the effects of pregnancy and baseline exposures on school-age outcomes ^19^, exploiting the rich SHINE dataset to control for confounding by pregnancy-related factors ( *e.g.*, wealth, maternal education, haemoglobin, maternal capabilities), the trial interventions, and the time-varying nature of exposures experienced between birth and 18 months of age.

***CHEU and CHU comparison (Objective 3d)*.** Baseline characteristics between CHEU and CHU groups will be compared using multinomial and ordinal regression models and Somers’ D for medians, while handling within-cluster correlation with robust variance estimation. We will use GEE models to compare each functional outcome, assessed using the SAHARAN Toolbox, between CHEU and CHU groups. A secondary adjusted analysis will be performed including possible confounders as defined in the SAP. Poisson regression will be used to compare the cumulative incidence of illness episodes, clinic visits, and hospitalizations between groups during prospective surveillance measured by the CHW illness questionnaire.

***Comparison between aspects of school-age growth and function and contemporary conditions (Objective 4)*.** Internal consistency of outcomes across cognitive domains will be compared, including the primary outcome (KABC-II total score) and other cognitive measurements. Similarly, consistency of physical function measurements and their associations with growth will also be measured, as previously described ^12^. Univariable analysis will explore associations between school-age growth, cognitive and physical function ( Table 1 to Table 3) and current environmental, schooling, nurturing and caregiving exposures ( Table 4). We will use LASSO for GEE to explore the effect of contemporary covariates on 7-year outcomes. We will also apply hierarchical clustering of principal component scores to group children according to their school-age outcomes. We will explore univariable distributions (central tendencies, variance, prevalence) of school-age outcome variables within each identified cluster of children to gain further insights into trade-offs and prioritisation in growth and function ^20^.

**Table 4.** Caregiver questionnaire contemporary exposures. MICS, Multi-indicator cluster survey from UNICEF; HFIAS, Household Food Insecurity Assessment Scale; HDDS, Household Dietary Diversity Score; FCS, Food Consumption Score; HWISE, Household Water Insecurity Experiences Scale; ARV, Antiretroviral. (Note that WG & SDQ sections are included in cognitive outcomes).

	QUESTIONNAIRE	DOMAIN	MEASURES	RATIONALE
**Caregiver ** **questionnaire** ** (90 mins)**	**Demographics**	Household (HH) composition	Main caregiver, caregiver years of schooling, religion, head of household, breastfeeding duration	Nurturing, household demographics, caregiver education
	**Socioeconomic status (SES)**	SES score	Overall score	Socio-economic status
	**Schooling & COVID impact**	School engagement & attendance	Years & months of schooling, Attendance, Alternative learning (if not in school), books at home	Child Education
	**Child adversity scale**	Adversities	Overall score (note different weightings may be applied)	Measure of accumulated adversities
	**Child parent relationship scale**	Caregiver’s relationship with child	Overall score, Closeness, conflict subscales	Nurturing
	**MICS Child discipline score**	Caregiver’s relationship with child	Overall score	Nurturing
	**Edinburgh Postnatal** ** Depression Scale**	Maternal depression	Overall score	Depression
	**Gender norms**	Caregiver gender norms	Overall score, sub-score on specific questions (education & violence)	Maternal capabilities
	**Social support**	Caregiver social support	Overall score	Maternal capabilities
	**HFIAS, HDDS, FCS**	Food insecurity & dietary diversity	HDDS score HFIAS score FCS score	Food insecurity
	**H-FOOD**	Land use	Land use, food aid, other adversities	Food insecurity
	**HWISE, Water access**	Water insecurity & access	HWISE score, water volume, water usage	Water insecurity
	**HIV**	HIV status	ARV treatment, Date of diagnosis,	HIV treatment history

***Development of novel outcome metrics (Objective 5)*.** Novel outcome metrics will be developed based on SAHARAN toolbox measurements conducted in 250 HUU children in the standard-of-care arm. We will use these data to undertake a factor analysis of the primary and secondary cognitive SAHARAN variables to select the most informative sparse combination of tests for development of a cognition metric (COG-SAHARAN). We will undertake reliability and agreement analyses to compare the performance of the shortened, open-access tools with the KABC-II tool, which is a gold-standard assessment, but is costly, time-consuming, complex and proprietary. A factor and reliability analysis will inform the generation of the growth metric (GROW-SAHARAN) from all growth and physical function outcomes. GROW-SAHARAN will measure the child’s growth, body composition and physical function to holistically assess school-age children’s nutritional status. Further analyses could tailor the GROW-SAHARAN to focus on key indicators within specific areas such as physical function, body composition or chronic disease risk. This is a sufficient sample size to derive the COG-SAHARAN and GROW-SAHARAN metrics, based on previously published metrics ^21, 22^. As outlined above, we will employ factor analysis as a data reduction step to i) identify the underlying constructs that we are measuring, ii) evaluate which of the multiple sub-tests are driving the variability measured, and iii) determine whether all tests are required.

Standardisation exercises with the same children will allow us to test the reliability of the proposed measures using intra-cluster correlation coefficients and to measure Cronbach’s alpha. Finally, the relationship between these novel metrics will be investigated within a recognised conceptual framework that evaluates child function ^23^ ( Figure 3). A combined child metric (“SUB-SAHARAN”) will also be developed after derivation of COG- and GROW-SAHARAN, by exploring the relationships between the two metrics. All metrics will then be operationalised in the 1,000 CHU randomized to early-life IYCF or WASH interventions, and the performance of each metric compared to the more detailed analysis outlined in previous sections.

## Dissemination

Results of the follow-up study will be disseminated through community gatherings, building on recent screenings of a locally made film about the SHINE trial. We will also leverage the district Community Engagement Advisory Board, which comprises key stakeholders from the community. Findings will be disseminated to the academic community through open-access peer-reviewed journals and conference presentations. In addition, a short animation video describing the school-age assessment metrics will be created and published online, as well as workshops with key local and national partners including Ministry of Health and Child Care, United Nations Children's Fund (UNICEF), Food and Agriculture Organisation, World Food Program, Ag2Nut and the Agriculture, Nutrition and Health (ANH) academy. An earlier version of this article can be found on medRxiv (doi: https://doi.org/10.1101/2022.08.17.22278247). Statistical code and dataset will be available from the data repository on request, https://clinepidb.org/ce/app. A creative commons licence CC-BY 4.0 will be applied to the dataset.

## Ethics

All research procedures are conducted in accordance with the Declaration of Helsinki and according to International Conference on Harmonisation GCP guidelines. The Medical Research Council of Zimbabwe (MRCZ) approved the study protocol (MRCZ/A/1675, 21/02/21). Adverse events and serious adverse events that are related to any trial procedures during the follow-up visits are reported to MRCZ. Internal monitoring is conducted, and findings requiring corrective action are categorised as critical, major, or minor, with appropriate timelines for resolution. This follow-up study is registered with the Pan-African Clinical Trials Registry ( PACTR202201828512110, 24/01/22).

## Study status

The study has currently finished recruiting with 1,275 children enrolled (100 HIV unexposed, 275 exposed to HIV, of whom seven were HIV positive). Data cleaning and analysis is currently ongoing. In addition, the monthly morbidity questionnaires and the annual Sub-SAHARAN monitoring visits are currently being performed.

## Discussion

### The ‘missing middle’ of school-age child health and function

The SHINE follow-up study aims to characterize school-age health and function and provide important insights into the ‘missing middle’ of childhood. It is known that between ages 5–14 years, mortality is concentrated in sub-Saharan Africa ^24^, but measures of child growth and development have frequently been overlooked ^25^. Nevertheless, this life-stage remains crucial for growth to consolidate positive gains from early childhood. Height and BMI monitoring shows that children can experience negative trajectories of increasing BMI in response to adverse environmental conditions ^26^, increasing future non-communicable disease risk. Examining growth and development together is urgently needed to understand the longer-term impact of early-life exposures and interventions in relation to school-age growth and function ^6^. Few studies have confirmed whether early growth gains translate into long-term improvements in cognitive and physical function and, conversely, whether interventions showing little or no obvious effect at early ages may confer meaningful health and functional benefits in later childhood. It is also important to understand the contemporary risk and protective factors that undermine or accelerate progress, respectively ( Figure 2). School-age presents an opportunity to mitigate early disadvantages and consolidate early gains, thus providing sustainable benefits in development and longer-term health.

### Strengths and limitations of this study

Follow-up of the SHINE cohort at 7 years of age will determine whether nutrition interventions in the first 1,000 days that modestly increase linear growth can restore long-term function, and whether WASH interventions in the first 1,000 days have any impact on school-age outcomes, despite no effects on early-life growth. This will be achieved using the SAHARAN toolbox to measure growth, neurodevelopmental and physical health outcomes of school-age children, which has previously been extensively piloted. The study efficiently capitalizes on the factorial trial design to evaluate both interventions in the same children. The feasibility of a similar detailed battery in children has previously been demonstrated before in a cohort study of survivors of severe acute malnutrition ^27^. Limitations include the selection of only one of the two districts of the SHINE trial, as well as the inability to follow-up children who have moved out of Shurugwi district. The selection of children who have not been exposed to HIV is randomised to ensure balance between the SHINE intervention arms. However, we will try to follow all children exposed to HIV, which may lead to an imbalance in treatment arms for CHEU. Children living with HIV are too few in number to provide meaningful inferences, so they will form a separate case report. Finally, all families received a latrine following the 18-month primary endpoint visit, so the focus on interventions is restricted to early-life, whilst recognising the long period between early-life randomized interventions and school-age outcome assessment.

### Effects of HIV exposure on school-age outcomes

There remains an urgent need to clarify whether the health gap between CHEU and CHU in sub-Saharan Africa persists at school age, and to identify the biological, social and environmental exposures in early life that underpin these differences. There is a lack of long-term data: one study in Zambia found that the gap between CHEU and CHU growth outcomes had widened by 7.5 years of age ^28^, while a study of neurocognitive outcomes across five African countries ^29^ found no differences between CHEU and CHU groups, but did not compare language, which may be most predictive of future function ^29^. Other cohorts have reported poorer mathematic abilities and reduced IQ among CHEU in south-east Asia ^30^, but studies have focused on a limited range of outcomes. Therefore, applying the SAHARAN toolbox to provide concurrent assessments of growth, health, physical capacity and cognition will allow a holistic insight into the long-term effects of HIV, ART and other pregnancy exposures on health and human capital.

### Derivation and application of metrics

Rural Zimbabwe represents a setting of subsistence farming, food and water insecurity, gender inequity and vulnerability climate change. Measuring these environmental exposures, as well as other household and individual factors, enables their effects on school-age growth and function to be investigated. The SAHARAN toolbox takes approximately 4 hours to complete in the field, which is not feasible for widespread applicability. However, using these data to generate readily accessible and applicable shorter metrics will provide future tools that rapidly assess either school-age cognitive function (COG-SAHARAN), physical function and growth (GROW-SAHARAN), or both (SUB-SAHARAN). These metrics will be derived from indicators and tests previously used in other LMIC and hence should be widely applicable. We will provide a toolkit for operationalisation of the metrics in other contexts, allowing other research groups and programmes to adapt the tools prior to implementation.

## Conclusions

Follow-up of the SHINE trial provides a unique opportunity to comprehensively characterise the long-term response to early-life nutrition and WASH interventions, for both children exposed to HIV and those who have not been exposed to HIV. In this context, use of the SAHARAN toolbox provides a holistic outcome measurement, which is crucial to understand school-age trajectories across different functional domains and the long-term impact of exposures including interventions, risk and protective factors. Our study will inform future policy and programming approaches to stunting since demonstration of long-term functional benefits would motivate scale-up of IYCF in areas of high stunting or HIV exposure. Conversely, a lack of school-age benefits suggests that alternative approaches to stunting, poor neurodevelopment and their subsequent mitigating strategies are urgently needed. The metrics themselves also represent paradigm shifts in evaluation of nutrition-sensitive interventions in a broader context; they will provide assessment tools for school-age growth and non-communicable disease risk, with functional measures of physical health and human capital. It is only by generating both the measurement tools and long-term data that evidence can be translated into action.

## Data Availability

No data are associated with this article. Open Science Framework: SHINE 7-year Follow-up. https://doi.org/10.17605/OSF.IO/PHV83 ^16^ This project contains the following extended data: Study protocol Data collection tools Completed SPIRIT checklist Data are available under the terms of the Creative Commons Attribution 4.0 International license (CC-BY 4.0).
