# Supplementary material for: The Sanitation Hygiene Infant Nutrition Efficacy (SHINE) Trial: Protocol for school-age follow-up
Source: Wellcome Open Res. 2023 Jul 14;8:306. [Version 1] doi: 10.12688/wellcomeopenres.19463.1 (PMC10685067; doi:10.12688/wellcomeopenres.19463.1)
Supplement: Peer review history

# Reviewer report: Reviewer response for version 1

**DOI:** 10.21956/wellcomeopenres.21560.r63496

**Published:** 2023-11-28

**Authors:** Aaron Kobina Christian

**Affiliations:** 1 Regional Institute for Population Studies, University of Ghana, Legon-Accra, Accra, Ghana

The paper adequately examines the longterm effect of the SHINE trial. Such analysis is important to ascertain the sustainability and/or sustaining effect of nutrition interventions over a period of time. I recommend the paper to be approved with minor revisions.

1. The authors need to include the number of district the SHINE trail was implemented in. This will then make sense of the limitation of selecting only one of the district for this study.
2. The first paragraph of the discussion seems out of place for me. It discusses more of the rationale of the research rather than beginning straight with the contribution of the paper to current scholarship. More of the information should be in the paragraphs information needs to be in the introduction.

Is the study design appropriate for the research question?

Yes

Is the rationale for, and objectives of, the study clearly described?

Yes

Are sufficient details of the methods provided to allow replication by others?

Yes

Are the datasets clearly presented in a useable and accessible format?

Yes

Reviewer Expertise:

Maternal and Child nutrition, Population Health

I confirm that I have read this submission and believe that I have an appropriate level of expertise to confirm that it is of an acceptable scientific standard.

# Reviewer report: Reviewer response for version 1

**DOI:** 10.21956/wellcomeopenres.21560.r66033

**Published:** 2023-09-20

**Authors:** Renuka Jayatissa

**Affiliations:** 1 Faculty of Nutrition and Food sciences, International Institute of Health Sciences, Colombo, Sri Lanka; 2 Department of Nutrition, Medical Research Institute, Colombo, Sri Lanka

Overall it is very good and comprehensive protocol and many researchers can replicate it. It is useful for comparison purposes. It is a fair amount of work by the authors and congratulate them for this hard work. This protocol will provide good resources to scientific community.  

- Introduction - Line 1 - Stunted is defined as HAZ less than - 2SD not more than. Need the correction.
- Limitation - Current Food security situation is not assessed, which is one of the key factors for school outcomes, better to add it. Birth weight is one of the key variable of stunting and school outcome, is it included in the analysis? If not better to include under design or limitations.
- Details on data tools - It is not fully available in this protocol, however, Previous data tools are available with the previous study publication. New component could not found. 

Is the study design appropriate for the research question?

Yes

Is the rationale for, and objectives of, the study clearly described?

Yes

Are sufficient details of the methods provided to allow replication by others?

Yes

Are the datasets clearly presented in a useable and accessible format?

Not applicable

Reviewer Expertise:

Public health nutrition, clinical nutrition, public health and migrant health

I confirm that I have read this submission and believe that I have an appropriate level of expertise to confirm that it is of an acceptable scientific standard.
